# Rational Design of CRISPR/Cas12a-RPA Based One-Pot COVID-19 Detection with Design of Experiments

**DOI:** 10.1021/acssynbio.1c00617

**Published:** 2022-04-01

**Authors:** Koray Malcı, Laura E. Walls, Leonardo Rios-Solis

**Affiliations:** † Institute for Bioengineering, School of Engineering, University of Edinburgh, Kings Buildings, Edinburgh EH9 3BF, United Kingdom; ‡ Centre for Synthetic and Systems Biology (SynthSys), University of Edinburgh, Kings Buildings, Edinburgh EH9 3BD, United Kingdom; § School of Natural and Environmental Sciences, Newcastle University, Newcastle upon Tyne NE1 7RU, U.K.

**Keywords:** one-pot COVID-19 testing, CRISPR/Cas12a, molecular diagnosis, definitive screening design, reaction optimization, recombinase polymerase amplification (RPA)

## Abstract

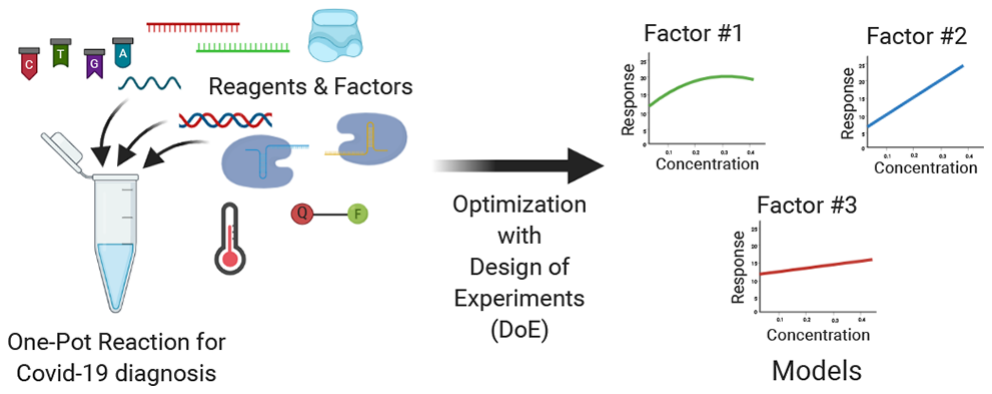

Simple and effective molecular diagnostic methods have gained importance due to the devastating effects of the COVID-19 pandemic. Various isothermal one-pot COVID-19 detection methods have been proposed as favorable alternatives to standard RT-qPCR methods as they do not require sophisticated and/or expensive devices. However, as one-pot reactions are highly complex with a large number of variables, determining the optimum conditions to maximize sensitivity while minimizing diagnostic cost can be cumbersome. Here, statistical design of experiments (DoE) was employed to accelerate the development and optimization of a CRISPR/Cas12a-RPA-based one-pot detection method for the first time. Using a definitive screening design, factors with a significant effect on performance were elucidated and optimized, facilitating the detection of two copies/μL of full-length SARS-CoV-2 (COVID-19) genome using simple instrumentation. The screening revealed that the addition of a reverse transcription buffer and an RNase inhibitor, components generally omitted in one-pot reactions, improved performance significantly, and optimization of reverse transcription had a critical impact on the method’s sensitivity. This strategic method was also applied in a second approach involving a DNA sequence of the N gene from the COVID-19 genome. The slight differences in optimal conditions for the methods using RNA and DNA templates highlight the importance of reaction-specific optimization in ensuring robust and efficient diagnostic performance. The proposed detection method is automation-compatible, rendering it suitable for high-throughput testing. This study demonstrated the benefits of DoE for the optimization of complex one-pot molecular diagnostics methods to increase detection sensitivity.

An effective test-and-trace system is one of the essential elements for the containment of the COVID-19 pandemic caused by the SARS-CoV-2 virus. Reverse transcription–polymerase chain reaction (RT-PCR), a method employed to amplify viral genetic material, is the most widely used approach for COVID-19 nucleic acid detection and is considered the gold standard thanks to its relatively high accuracy.^1,2^ However, as RT-PCR relies on expensive equipment and specially trained personnel, testing is typically performed in centralized laboratories by experts.^3^ This necessitates the transportation of samples to dedicated test centers, causing delays in sample analysis and result dissemination. For more effective and streamlined testing, rapid and reliable diagnostic methods that can be performed without extensive training and expensive equipment, such as thermal cyclers, are critical.^4^

Inexpensive lateral flow devices have been widely used for mass viral antigen testing as a result of their inherent simplicity and ease of use. However, lateral flow tests are significantly less reliable than nucleic acid-targeting methods due to their reduced specificity and sensitivity.^5^ Nucleic acid-based methods also offer the advantage of variant-specific detections. For instance, the most recent threat, variant B.1.1.529, more widely known as Omicron, contains unprecedented mutations on the spike protein.^6^ In such cases, nucleic acid-based methods can be more advantageous.

In the past few decades, several isothermal nucleic acid amplification methods have been developed including transcription-mediated amplification,^7^ loop-mediated isothermal amplification,^8,9^ and recombinase polymerase amplification (RPA).^10^ Using such methods, as the target fragments can be amplified at a constant temperature, the need for advanced thermal cyclers is eliminated. Among them, RPA utilizes recombinase/primer complexes to scan target DNA regions on the template resulting in strand exchange. Following that, a DNA polymerase amplifies the target fragment.^10^ Similar to RT-PCR, reverse transcription can be combined with RPA, named RT-RPA, to amplify a region of interest in an RNA template.^11,12^ Recently, RT-RPA has been coupled with CRISPR systems, and varied methods have been proposed for COVID-19 molecular diagnosis as alternatives to RT-PCR.^13,14^

For example, using a Cas12a from *Lachnospiraceae bacterium* ND2006 (LbCas12a) following an RT-RPA reaction, which amplified the *ORF1ab* region of COVID-19 genome, Curti et al. (2020) successfully detected 10 copies/μL of viral RNA.^15^ Ding et al. (2020) also recently developed a one-pot reaction system, termed “All-In-One Dual CRISPR-Cas12a (AIOD-CRISPR)”, involving two LbCas12a/gRNA complexes targeting two different locations on the template.^16^ Using this RT-AIOD-CRISPR system, the researchers were able to detect 5 copies/μL of N gene using blue-LED or UV light.^16^ Thanks to their simplicity and ease of use, one-pot reactions are drawing attention as potential point-of-care testing alternatives.^16−18^ In a typical one-pot reaction system, however, many reactions involving multiple factors and interactions take place simultaneously. Therefore, elucidating which of the many factors have a significant effect on performance and subsequently determining the optimal settings for each can be extremely difficult and/or time-consuming using the traditional approach of changing one factor at a time.^19^ This is problematic as optimization of the reaction has the potential to translate into important economic savings. Design of Experiments (DoE), a strategic approach allowing the systematic exploration of complex systems, can be implemented for the optimization of biological systems.^20,21^

Furthermore, as DNA templates are often used to optimize one-pot reactions containing a reverse transcription step,^16,22,23^ suboptimal conditions may be selected for methods involving RNA templates as reaction conditions differ according to the template used. DoE is a useful tool for efficient reaction-specific optimization, as a large number of factors can be screened simultaneously, allowing the optimal conditions for each reaction to be determined using relatively few experimental runs.

In addition to the efficiency of diagnostic testing methods, their scalability is essential for the accurate determination of the number of individuals within a population who are currently infected. Through the use of automation and standardization, throughput, accuracy, and reproducibility among test centers can be increased dramatically. As a result, a number of COVID-19 molecular diagnostic methods have been partially or fully automated for high-throughput testing.^24,25^ In addition, a novel mobile testing center was recently developed within a shipping container, making use of five open-source, affordable Opentrons OT-2 automation platforms for liquid handling, and the researchers were able to perform up to 2400 tests per day.^26^ In addition to being relatively affordable and high-throughput, these platforms provide the additional benefit of being readily transportable. The development of new low-cost and effective COVID-19 testing methods that are automation-compatible is therefore critical for large-scale testing.

In the present study, a low-cost, automation-compatible, one-pot CRISPR-based COVID-19 diagnostic method was developed. Strategic three-level definitive screening designs were employed to elucidate key factors with a significant effect on performance, and statistical models were derived to determine the optimal settings of such factors, to maximize test sensitivity and detection capacity. The proposed one-pot COVID-19 detection method consisted of three distinct reactions: (1) reverse transcription of the SARS-CoV-2 RNA to generate complementary DNA, (2) amplification of the resulting DNA using a recombinase polymerase amplification, and (3) perform collateral activity on a reporter probe using CRISPR/Cas12 in the presence of the target DNA fragment; these reactions are summarized in Figure 2.

The unique features of Cas12a (formerly Cpf1) play a critical role in this method. In contrast to Cas13a (formerly C2c2) targeting RNA sequences,^27^ Cas12a targets DNA sequences, and an RNA-guided Cas12a can bind to the 20 bp ssDNA target sequence and trigger activation without the need for protospacer adjacent motif (PAM) to perform nonspecific single-stranded DNase (ssDNase) activity, even though PAM is needed for cleavage of a dsDNA substrate.^28^ The ssDNA arising during RPA reaction, at the strand displacing and/or polymerization,^10^ acts as an activator for Cas12a. Subsequently, ssDNA-FQ reporters in the reaction mix are cut by activated Cas12a and produce fluorescent signals as a response to the presence of target nucleic acid. It has been shown that the use of two gRNAs targeting two different regions on the templates enhances fluorescent emission sufficiently for visual detection, even with low copy numbers.^16^ To enhance the fluorescent signals emitted by the fluorescein (6-FAM) used in the assay of this study, two gRNAs were employed to simultaneously target the amplicons. The same principle was also used for DNA templates, except RNA-related reagents such as reverse transcriptase were omitted, to investigate whether template-specific optimization is necessary.

In this study, the design of experiments guided reaction optimization improved assay sensitivity, facilitating the detection of just two copies of the COVID-19 RNA genome and 0.5 copies of the COVID-19 DNA fragment per μL. These are among the lowest copy numbers that have been detected to date using CRISPR/Cas12 technology coupled with RPA for isothermal nucleic acid amplification. The benefits of statistical design of experiments for efficient optimization of molecular diagnostic methods involving complex biological reactions were therefore demonstrated.

## Materials and Methods

### Nucleic Acids, Reagents, and Kits

Primers, single-stranded DNA fluorophore-quencher (ssDNA-FQ) reporter containing 6-Carboxyfluorescein (6-FAM) at 5′ end, and gRNAs were ordered from IDT. Synthetic DNA fragment (300 bp) of N gene was ordered from Twist Bioscience. Full-length SARS-CoV-2 genomic RNA (AcroMetrix Coronavirus 2019 RNA Control, RUO) was ordered from Thermo Fisher Scientific. A yeast plasmid, p426_Cas9_gRNA-ARS511b, was purchased from Addgene and was used as nonspecific DNA control (NSDC). RPA kit (TwistAmp Basic) was ordered from TwistDX. *Lachnospiraceae bacterium ND2006* Cas12a (EnGen Lba Cas12a), M-MuLV reverse transcriptase, murine RNase inhibitor, and NEBuffer 2.1 were ordered from New England Biolabs (NEB). GeneJET PCR Purification Kit was purchased from Thermo Fisher Scientific. The list of nucleic acids used in the study can be found in Table S1.

### RT-RPA-CRISPR Assays

Before starting diagnosis assays, the RPA kit was tested to determine whether the manual mixing during incubation, which is recommended by the supplier (TwistDX), is essential. Two RPA primer pairs targeting the N gene (Table S1) were used and different test conditions, manually mixed and unmixed, were compared on agarose gel after 30 min incubation at 39 °C as shown in Figure S1. RPA products were purified by using GeneJET columns and the yields of RPA products were quantified by using NanoDrop Spectrophotometer (Thermo Fisher Scientific).

Stock solutions of Cas12a/gRNA complex were prepared by mixing Cas12a, gRNA1, and gRNA2 at concentrations of 10 μM in 1× NEBuffer 2.1. The resulting mixtures were incubated for 15 min at room temperature before use. The Cas12a/gRNA stock solution was then stored at −20 °C until further use. For RT-RPA-based assays, 2.4 μL forward RPA primer (10 μM) and 2.4 μL reverse RPA primer (10 μM) were added into 29.5 μL rehydration buffer. Following that, M-MuLV reverse transcriptase, reverse transcriptase buffer, murine RNase inhibitor, ssDNA F-Q reporter (reporter DNA), and water were added into the rehydration buffer with various concentrations as discussed below. This mix was then used to resuspend the enzyme pellet provided with the RPA kit. Following resuspension of the enzyme pellet, Cas12a/gRNA complex and MgOAC (20× diluted in final volume) were added respectively to the solution. Template RNA was added after resuspending the enzyme pellet when different conditions were tested; for sensitivity tests, it was added directly to the rehydration buffer. For RPA-based assays containing DNA template, this protocol was used without the addition of M-MuLV reverse transcriptase, reverse transcriptase buffer, and murine RNase inhibitor.

### Fluorescence Detection

The fluorescence generated by the DNA reporter was measured using a CLARIOstar Plus microplate reader (BMG LABTECH). At the end of the definitive screening design runs, 20 μL reaction volume was mixed with 80 μL water in each well of a black and clear flat-bottom 96-well microplate (Greiner). For sensitivity tests, 1 μL reaction samples were taken and measured every 10 min. To measure the fluorescent intensity of 6-carboxyfluorescein (6-FAM), the excitation wavelength was set to 495 nm and the emission wavelength was set to 520 nm with an 8 nm bandwidth for both. An enhanced dynamic range (EDR) was used for fluorescence gain. To visually observe the test tubes, UV light (BioDoc-It, UVP) and a blue-LED transilluminator (Safe Imager 2.0, Thermo Fisher Scientific) were used.

### Experimental Design, Analysis, and Software

To test the effects of different factors in RT-RPA-CRISPR assays, JMP data analysis software (SAS) was employed for both design of experiments and statistical modeling. A three-level definitive screening design (DSD) capable of screening second-order effects^29^ was used for experimental designs. The numerical values of fluorescence intensity obtained from the microplate reader were used as a response in the designs. The order of the conditions was randomized and a minimum of 2*n* + 1 conditions, where “*n*” represents the factor number, was tested. Taking advantage of the randomization in DSD, each condition was tested once with 2*n* + 1 total conditions for each experiment. Forward stepwise regression^30^ was used to make the models with minimum Bayesian information criterion (BIC)^31^ as a stopping rule for stepwise regression control. To find the optimum value for each factor, the desirability score was maximized, and the parameters suggested by the models were used to find the lowest possible copy numbers that can be detected by (RT-)RPA-CRISPR assays. The sensitivity experiments to detect ultralow copy numbers of DNA and RNA templates were conducted in at least duplicate, and one-way analysis of variance (ANOVA) was used to determine whether there were any statistically significant differences between different copy numbers and the controls. Nontemplate control (NTC) and nonspecific DNA control (NSDC) containing the plasmid p426_Cas9_gRNA-ARS511b were used as control reactions. The error bars indicate the standard deviations within the samples. The illustrations were made using Biorender.

## Results and Discussion

### Amplification-Free Detection Using Only Reverse Transcription and Cas12a/gRNA Complexes

**Figure 1:**
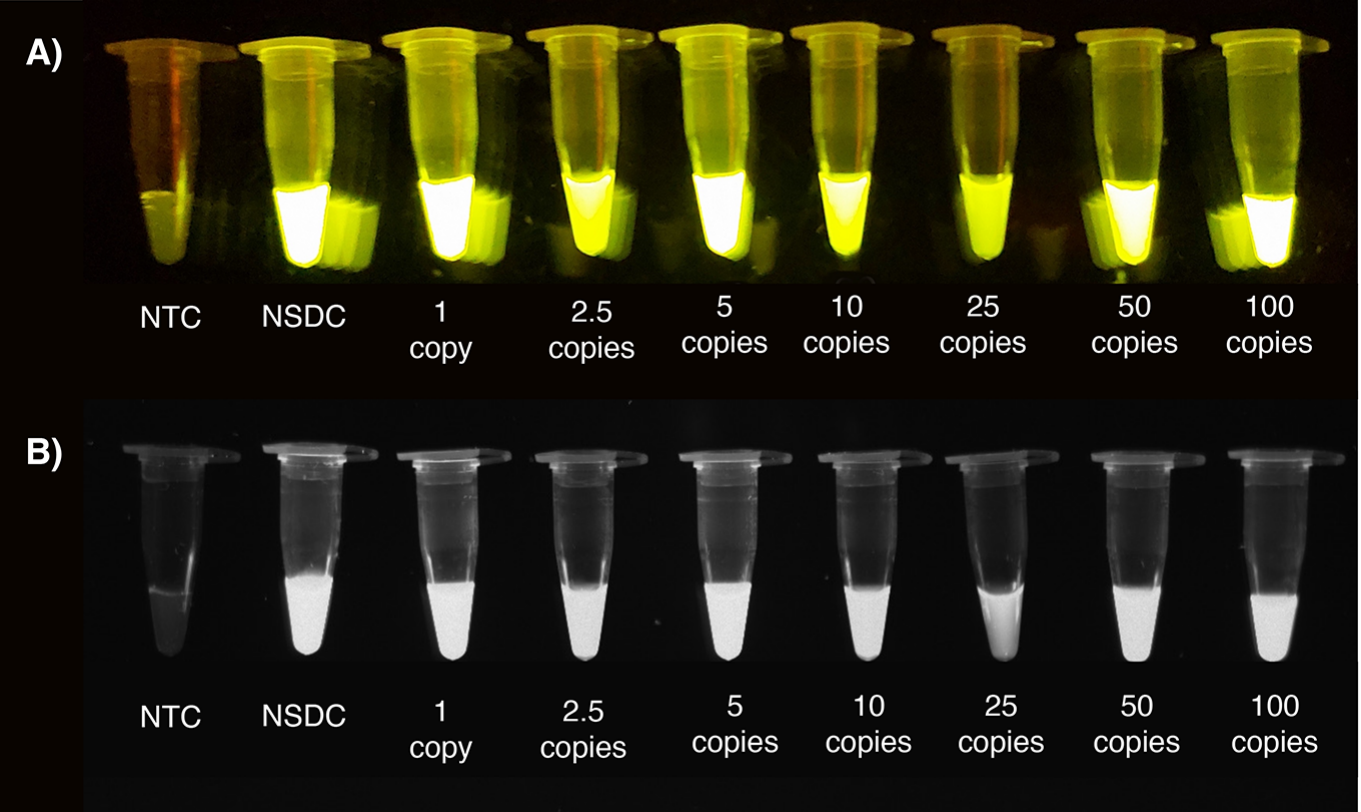
Fluorescence emissions from the tubes containing cDNA products from different copy numbers of SARS-CoV-2 genome after reverse transcription reaction followed by CRISPR/Cas12a assay. (A) On the transilluminator. (B) Under the UV light. NTC: nontemplate control, NSDC: nonspecific DNA control.

The sensitivity of the Cas12a/gRNA complex has been found to be relatively high, as it is capable of acting on just a few copies of DNA targets.^32−34^ It was therefore hypothesized that amplification of the DNA templates, resulting from reverse transcription of the SARS-Cov-2 RNA, may not be necessary. To investigate this, a standard 20 μL reverse transcription reaction^35^ was performed using the full-length SARS-CoV-2 genome. A 2 μL aliquot of the resulting product was subsequently used as a DNA template for a CRISPR assay containing the Cas12a/gRNA complexes and ssDNA F-Q reporter. Interestingly, after 45 min of incubation at 37 °C, strong fluorescence emissions were observed from all copy numbers ranging from one copy/μL to 100 copies/μL as shown in Figure 1. However, fluorescence emissions were also observed in a nonspecific DNA control (NSDC) assay, which was run in parallel as shown in Figure 1. Although no emission was detected in the nontemplate control (NTC, Figure 1), similar emission levels were detected in all five experimental replicates in the case of the NSDC. This highlighted that omission of the amplification step may result in low specificity, if nonspecific nucleic acids are present within the reaction, despite the extremely high sensitivity. This is particularly problematic for clinical applications, where contaminating nonspecific nucleic acids could be present in the samples. Further research is needed to understand the mechanisms behind these false positives and facilitate the use of amplification-free, specific, and ultrasensitive detection methods. In order to increase the specificity of the one-pot assay of this study, all subsequent experiments incorporated an amplification step with RPA to ensure fluorescence levels resulting from the DNA fragments of interest would be significantly greater than those resulting from contaminants within the samples.

### RPA Test

In standard RPA reactions, a manual mixing step during the incubation is recommended by the manufacturer, TwistDX, to maintain a homogeneous reaction environment and minimize substrate localization.^36^ However, manual handling is an obstacle for automated high-throughput testing and the incorporation of shaking apparatus is undesirable as it would necessitate increased capital investment. For this reason, the feasibility of omitting the manual mixing step was investigated. The forward and reverse RPA primers (Table S1) targeting a 121 bp region on the N gene and the forward and reverse RPA test primers (Table S1) targeting a 237 bp region on the same gene were used for RPA reactions. While the correct bands were observed from both mixed and unmixed reactions (Figure S1), indicating a successful reaction, significantly more DNA product (*p* < 0.01) was obtained during the mixed reactions, irrespective of the primer pairs used (Figure S2). Although the incorporation of an automated mixing step would be feasible even with basic liquid handling platforms, in addition to the increased costs it would incur, intervention during the amplification process could possibly cause cross-contamination if multiple samples were handled simultaneously.^37^ As successful amplification was achieved in the absence of mixing albeit with reduced efficiency, a decision was made to omit the mixing step in the reactions of this study to maximize cost-effectiveness and minimize contamination risks.

### One-Pot RT-RPA-CRISPR Reaction

**Figure 2:**
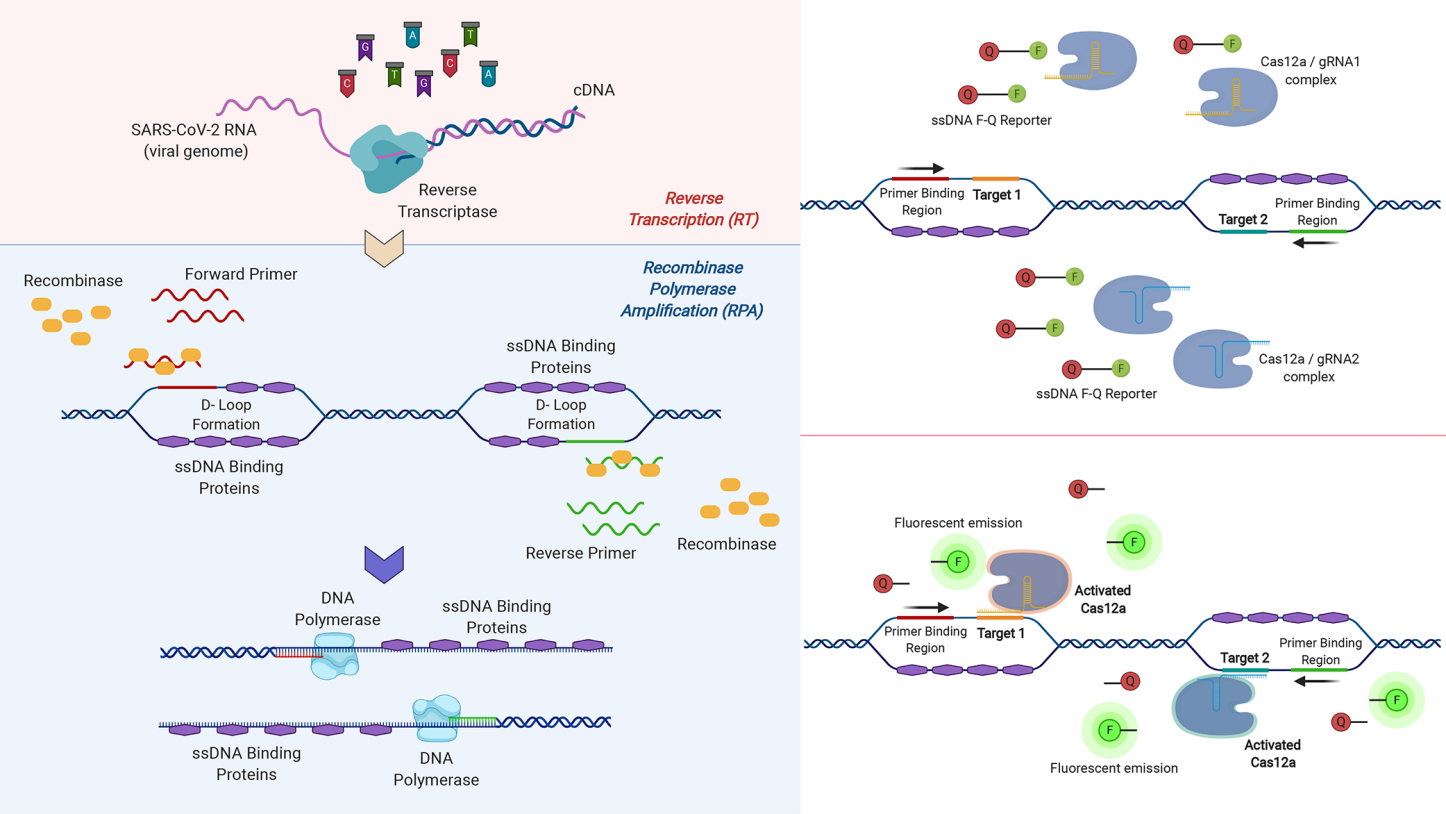
Working mechanism of the one-pot RT-RPA-CRISPR detection method. (A) With reverse transcription (RT), the target region on the viral genome is converted to cDNA by reverse transcriptase (M-MuLV). Following this, recombinases form a complex with forward and reverse primers and move them toward their homologous sequences on the template DNA, and this triggers strand displacement. ssDNA binding proteins stabilize the displaced strands resulting in D-loop formation. After this, DNA polymerase binds to the template and synthesizes DNA. With repeated cycles of recombinase polymerase amplification (RPA), the target DNA sequence is amplified. (B) Two different Cas12a/gRNA complexes targeting two distinct regions on the amplicons are also found in the environment. During RPA reaction, ssDNA arises allowing Cas12a/gRNA complexes to bind to their targets. (C) ssDNA targets of Cas12a/gRNA complexes act as activators so that activated Cas12a performs nonspecific ssDNase activity and cuts ssDNA F-Q reporters. The fluorescent tags (F), 6-FAM, get free from quenchers (Q), and then create fluorescence signals as a response to these reactions. These three reactions following each other continue simultaneously after several cycles.

As outlined in more detail in the introduction, the one-pot COVID-19 detection method used in this study involved three sequential reactions: reverse transcription (RT), recombinase polymerase amplification (RPA), and CRISPR/Cas12 assay. The target region of the cDNA produced during the RT reaction is amplified in the subsequent RPA reaction, yielding ssDNA. The specific region of this ssDNA is then recognized by Cas12a/gRNA complex, triggering binding and activation of Cas12a, which causes cutting of the reporter DNA and production of the fluorescence signal. This cycle of reactions iterates under isothermal conditions. The steps involved in this one-pot detection method are illustrated in detail in Figure 2.

### The First Round of Definitive Screening Design (DSD) for RT-RPA-CRISPR

The proposed one-pot detection method, involving three separate reactions, has many factors with the potential to influence performance as shown in Figure 2. To maximize the sensitivity and detection capacity of the method, while also minimizing costs through avoiding excess reagent use, these reactions should be optimized. It was also hypothesized that optimization may lead to sufficient improvements in selectivity to facilitate the detection of ultralow copy numbers. The effect of key factors on the fluorescent response was therefore investigated in detail using a statistical three-level definitive screening approach. The first step involved the reverse transcription of the RNA template, using reverse transcriptase, to synthesize cDNA. In a standard reverse transcription protocol, the use of an RNase inhibitor and dithiothreitol (DTT) was recommended by the manufacturers.^35,38^ The RNase inhibitor is used to block any RNase activity resulting from contamination in the reaction mix,^39^ and DTT is a reducing agent for disulfide bonds used to stabilize enzymatic activity and indirectly preserve the RNA template.^40^ However, the use of RNase inhibitor and/or RT buffer containing DTT and other compounds for a suitable reaction condition is generally omitted in one-pot RT-RPA-based detection reactions.^16,17,41^ Therefore, RNase inhibitor and RT buffer were included as factors in the screening design to investigate whether their addition could have a positive effect on the response (relative fluorescence unit, RFU). Increasing the quantity of dNTP is recommended by the manufacturer, to improve the efficiency of the reverse transcription step.^42^ As DNA polymerization reactions occur in both the RT and RPA steps, in this case, the dNTP concentration was also deemed important. As previous studies have also demonstrated that the Cas12/gRNA and ssDNA-FQ reporter concentrations can influence the resulting fluorescent intensity,^16^ these were also included as factors in the design. In addition to the aforementioned reagents and enzymes, additional variables including reaction volume, incubation time, and temperature were also considered. The temperature was of particular interest as the optimum working temperatures for the reaction enzymes have been reported to be between 37 to 42 °C. An eight-factor definitive screening design (DSD) was created using JMP Pro 14 statistical software to investigate the effect of each of the factors of interest in the fluorescent response as summarized in Table 1. For each test condition, 5 copies/μL of full-length COVID-19 RNA genome were used as a template. The results of each of the 17 treatments included in the experimental design are also summarized in Table 1.

**Table 1:** Experimental Design and the Fluorescence Responses (RFU) Obtained from the First Round of Definitive Screening Design (DSD)

condition	dNTP mix (mM)	RNase inhibitor (U/μL)	RT buffer (X)	Cas12a/gRNA (nM)	reporter DNA (μM)	reaction volume (μL)	incubation time	temperature (°C)	relative fluorescence unit (RFU)
**1**	0	0.8	1	25	0.2	50	90	39.5	**1494**
**2**	0.5	0	0	1000	10	20	30	39.5	**21 272**
**3**	0.5	0.8	0.5	1000	10	50	90	42	**56 820**
**4**	0	0	0	1000	0.2	35	90	42	**6746**
**5**	0	0	1	512.5	10	50	30	42	**23 604**
**6**	0.5	0.8	0	512.5	0.2	20	90	37	**3173**
**7**	0.5	0.8	1	25	10	35	30	37	**22 278**
**8**	0.25	0.8	1	1000	0.2	20	30	42	**1145**
**9**	0.5	0.4	0	25	0.2	50	30	42	**1141**
**10**	0	0	0.5	25	0.2	20	30	37	**1296**
**11**	0.5	0	1	25	5.1	20	90	42	**16 388**
**12**	0	0.4	1	1000	10	20	90	37	**29 634**
**13**	0	0.8	0	1000	5.1	50	30	37	**44 811**
**14**	0.25	0.4	0.5	512.5	5.1	35	60	39.5	**33 805**
**15**	0	0.8	0	25	10	20	60	42	**12 589**
**16**	0.25	0	0	25	10	50	90	37	**19 575**
**17**	0.5	0	1	1000	0.2	50	60	37	**2366**

**Figure 3:**
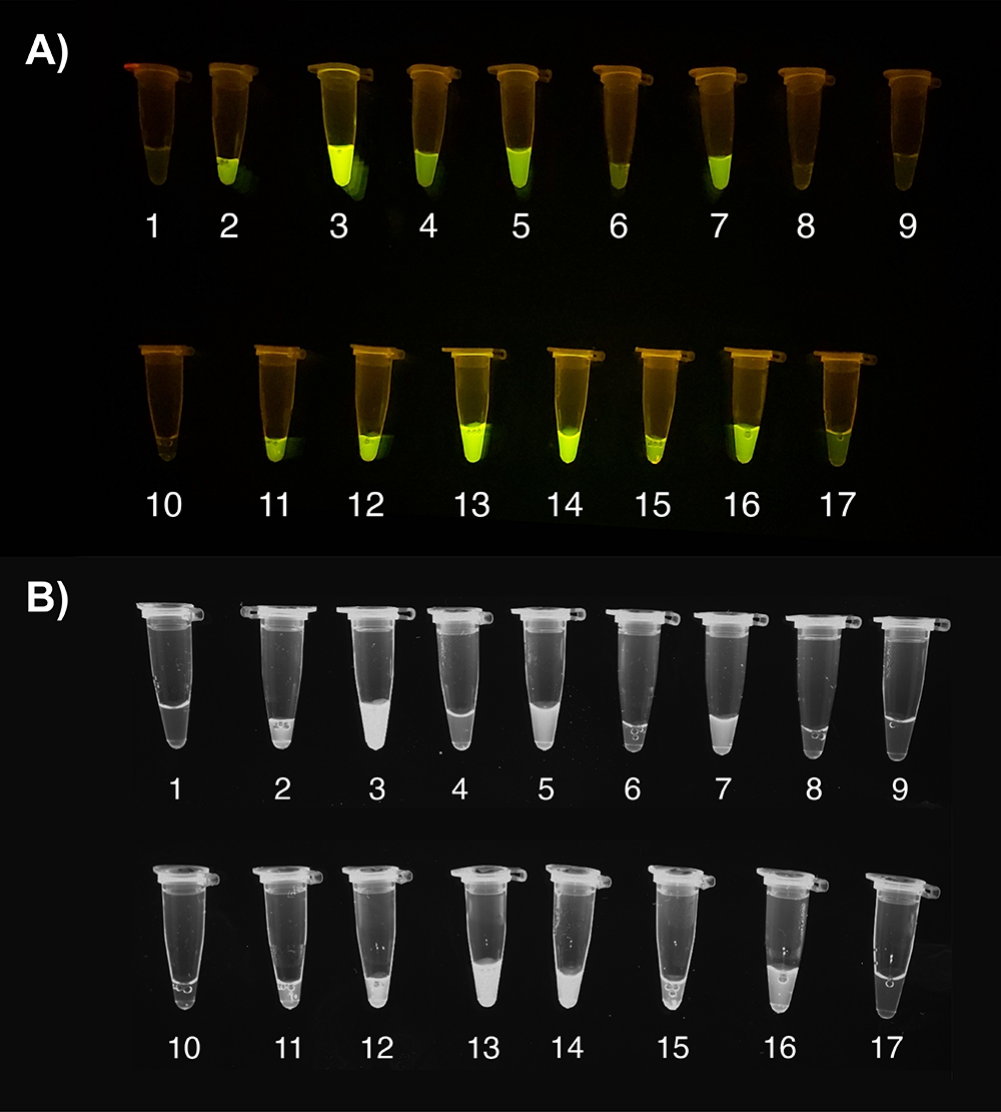
Visual comparison of each condition tested in the first definitive screening design. The numbers represent each condition as shown in Table 1. (A) On a blue-LED transilluminator. (B) Under UV light.

The fluorescence of the one-pot reaction tubes resulting from each of the 17 treatments was also visualized using a blue-LED transilluminator and under UV light (Figure 3). As expected, a strong correlation between the brightness of the tubes and the recorded fluorescence signals was observed, as shown in Figure 3.

**Figure 4:**
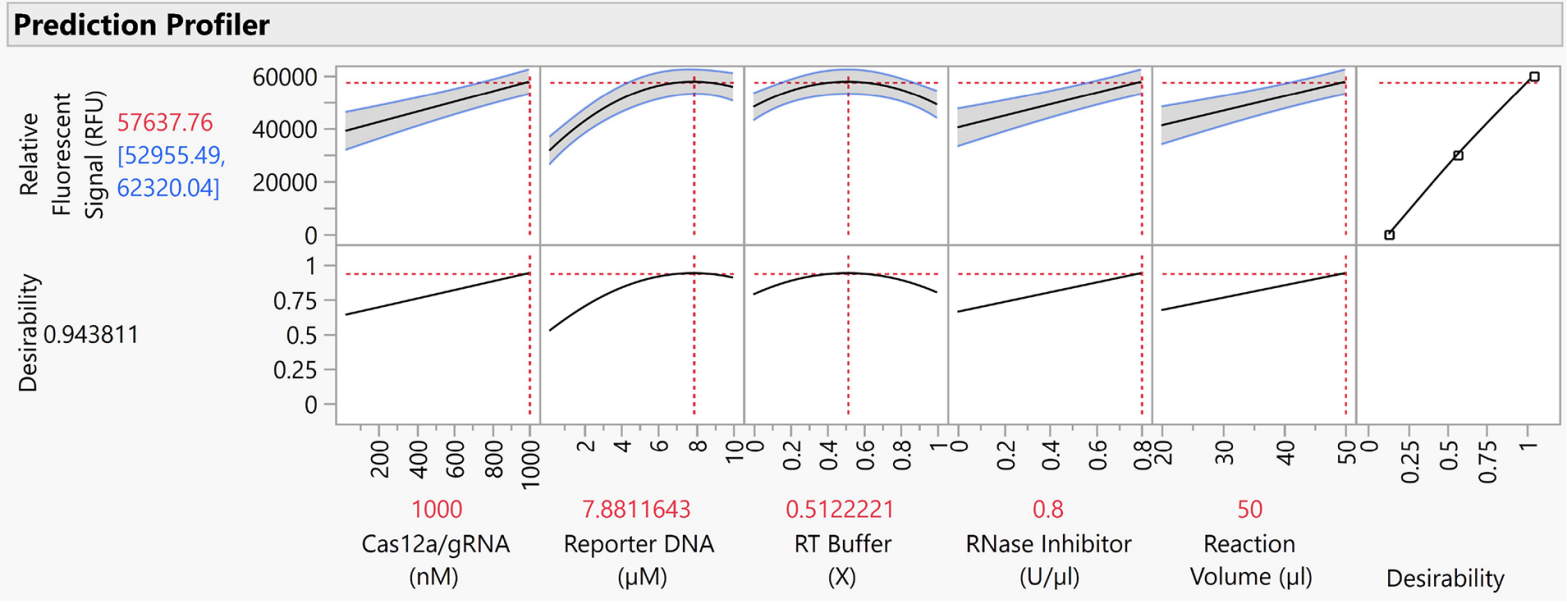
Models shown in the prediction profiler of the software (JMP). The substantial factors and their effects are shown with the maximized desirability score (0.94), reflecting the optimum parameters to obtain the highest fluorescent signal (RFU). The response (or desirability) is shown on the *Y*-axis and the factors are shown on the *X*-axis. The blue numbers shown on the response represent the minimum and maximum responses that can be obtained with the optimum parameter of each factor, while the red number represents the mean of the blue numbers. The gray areas between the blue lines represent the confidence interval for each plot. The plots at the bottom show the maximum desirability when the optimum parameter of each factor is used.

The effect of the eight factors of the DSD (Table 1) on fluorescent intensity was evaluated by forward stepwise regression using JMP and the BIC stopping criterion. A full quadratic model was derived, thereby considering all main effects and any second-order interactions. The resulting statistical model revealed Cas12a/gRNA (*p* < 0.001), reporter DNA (*p* < 0.001), RT buffer (*p* < 0.001), and RNase inhibitor concentrations, along with reaction volume (*p* < 0.001) were significant main effects. The statistical model was subsequently used to predict the optimal settings for each of these significant factors as summarized in Figure 4.

Interestingly, the dNTP concentration and reaction temperature did not have a significant effect on performance. As a result, the dNTP concentration of the kit was found to be suitable, and no additional supplementation was deemed necessary. Similarly, as the RT-RPA-CRISPR reaction was not significantly affected by temperature, it could be performed at the lower temperature of 37 °C to minimize energy requirements. Although the incubation time did not meet the criteria for incorporation into the statistical model, as its *p*-value was only just above the threshold at 0.055, this factor was subjected to further independent study, as speed can be an important factor on large scale diagnostic assays. It was therefore decided to perform further kinetic investigation through taking intermediate measurements throughout the incubation as further discussed in the following section. The concentration of the Cas12a/gRNA complex had a linear relation with the response, with increasing concentrations leading to increased fluorescence. This was expected as with increased Cas12a/gRNA complex availability, more cuts can be made in the ssDNA F-Q reporters per unit time generating a stronger fluorescent signal. The effect of ssDNA F-Q reporter (reporter DNA) concentration was nonlinear with an optimum concentration of ∼7.9 μM (Figure 4B). Up to this concentration, increasing reporter DNA concentration improved the response; however, at concentrations above ∼7.9 μM, no further improvements in fluorescence can be expected. This was likely the result of saturation of the Cas12a/gRNA complexes, as a result, a reporter DNA concentration of around 7.9 μM was deemed optimal to avoid the use of redundant reagents and minimize the reaction cost.

Most interestingly, the addition of RT-buffer and RNase inhibitor, factors which are generally neglected for one-pot RT-RPA-CRISPR reactions, had a significant effect on fluorescence. RT-buffer had a nonlinear relationship with the response, showing that a buffer concentration of ∼0.5× is optimal for the one-pot reaction. The response was reduced when lower or higher RT-buffer concentrations were used (Figure 4). RT-buffer provides a supply of DTT and KCl, which are not typically present in typical one-pot reactions. Although a concentration of 1× is typically recommended for optimal reverse transcriptase activity, in the complex environment of the one-pot reaction a 0.5× concentration yielded a higher fluoresce intensity. Higher RFU was obtained with increasing RNase inhibitor concentration, likely due to the preservation of the RNA template during the reaction, as unwanted contaminants may be present in this crowded environment, especially sourced by the enzyme pellet in the RPA kit.^36^ Finally, a reaction volume of 50 μL, which is equal to that of a standard RPA reaction, showed the best result.

### The Second Round of DSD for RT-RPA-CRISPR

**Figure 5:**
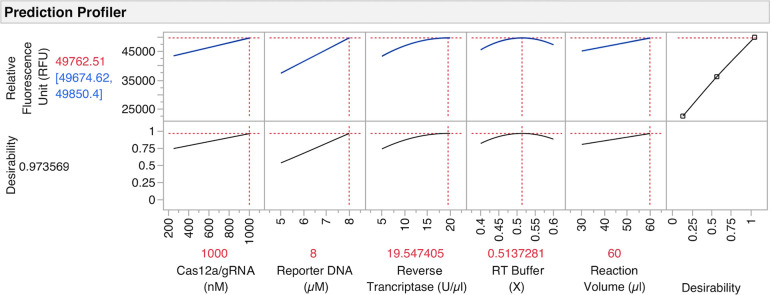
Models shown in the prediction profiler of the software (JMP). The substantial factors and their effects are shown with the maximized desirability score (0.97), reflecting the optimum parameters to obtain the highest fluorescent signal (RFU). The response (or desirability) is shown on the *Y*-axis and the factors are shown on the *X*-axis. The blue numbers shown on the response represent the minimum and maximum responses that can be obtained with the optimum parameter of each factor, while the red number represents the mean of the blue numbers. As the models show a good fit, the difference between the minimum and maximum responses is very small, so the confidence interval is not seen. The plots at the bottom show the maximum desirability when the optimum parameter of each factor is used.

The first DSD used to elucidate factors with a significant effect on the response and determine their optimal settings was extremely informative (Figure 5). Nevertheless, the second round of screening was performed to validate the results of the first DSD and further optimize those factors with the greatest influence on performance. To this end, the five significant factors elucidated during the first round of DSD screening were selected for further investigation. Using the results of the first study (Figure 4), more appropriate limits were determined for each factor. For example, in the first DSD, the concentration range of RT buffer was from 0× to 1×. As the optimum concentration was found to be ∼0.5×, its range was narrowed from 0.4× to 0.6× to find a more precise value. However, in the case of the Cas12a/gRNA complex an upper limit of 1000 nM (1 μM) was retained, as further increases would incur additional detection costs. In the second round of DSD an additional factor, reverse transcriptase concentration was also investigated. The experimental design and subsequent results are summarized in Table 2.

**Table 2:** Experimental Design and the Fluorescence Responses (RFU) Obtained from the Second Round of Definitive Screening Design (DSD)

condition	reverse transcriptase (U/μL)	RT buffer (X)	RNase inhibitor (U/μL)	Cas12a/gRNA (nM)	reporter DNA (μM)	reaction volume (μL)	relative fluorescence unit (RFU)
**1**	20	0.6	1	250	6.5	30	**31 001**
**2**	5	0.6	0.2	250	8	45	**32 949**
**3**	12.5	0.5	0.6	625	6.5	45	**37 197**
**4**	20	0.6	0.2	625	5	60	**29 862**
**5**	5	0.4	1	625	8	30	**31 530**
**6**	5	0.4	0.2	1000	6.5	60	**30 942**
**7**	5	0.5	1	250	5	60	**24 842**
**8**	20	0.4	1	1000	5	45	**31 575**
**9**	5	0.6	0.6	1000	5	30	**26 798**
**10**	12.5	0.4	0.2	250	5	30	**25 329**
**11**	12.5	0.6	1	1000	8	60	**45 872**
**12**	20	0.4	0.6	250	8	60	**38 289**
**13**	20	0.5	0.2	1000	8	30	**48 269**

As for the first DSD, the effects of each factor were evaluated via forward stepwise regression with the BIC stopping criterion. The second DSD successfully confirmed the results of the first DSD and facilitated the determination of the optimal settings with increased confidence as summarized in Figure 5. The confidence intervals generated by the model were very small, while the relatively narrow ranges of the factor levels likely contributed; this is indicative of model overfitting. Previous studies have found that due to the relatively low sample size of a 13-run DSD, the BIC fitting criterion can lead to overfitting of the model.^43^ To investigate this further, the model was rederived using an alternative fitting criterion, which has been found to perform better in certain cases for fitting DSD results, the Akaike information criterion corrected for small samples (AICc).^43,44^ Using the AICc criterion, the model contained three significant main effects, Cas12a/gRNA, reporter DNA, and reverse transcriptase as summarized in Figure S3. While the AICc model was smaller as expected, the optimal settings for the three active factors (Figure S3) were almost identical with those determined by the BIC model (Figure 5). According to the BIC model, increasing the reaction volume beyond 50 μL may increase fluorescence intensity slightly. It is well documented that reaction volume and topology have an impact on biochemical reactions.^45,46^ The results of the first DSD revealed that increasing the reaction volume to 50 μL was favorable for the one-pot reaction. However, as reaction volume was not deemed significant by the more stringent AICc model during the second round of screening and improvements predicted by BIC were relatively subtle (Figure 5), increasing the reaction volume beyond 50 μL may not be cost-effective. No significant effect was found in the narrowed concentration range of the RNase inhibitor; therefore, it was not included in the models of the second DSD. On the other hand, increasing reverse transcriptase concentration led to an improved fluorescence response, likely due to an enhanced yield of the upstream reverse transcription reaction, in the one-pot reaction.^42^

### DSD for RPA-CRISPR Using DNA Template

**Figure 6:**
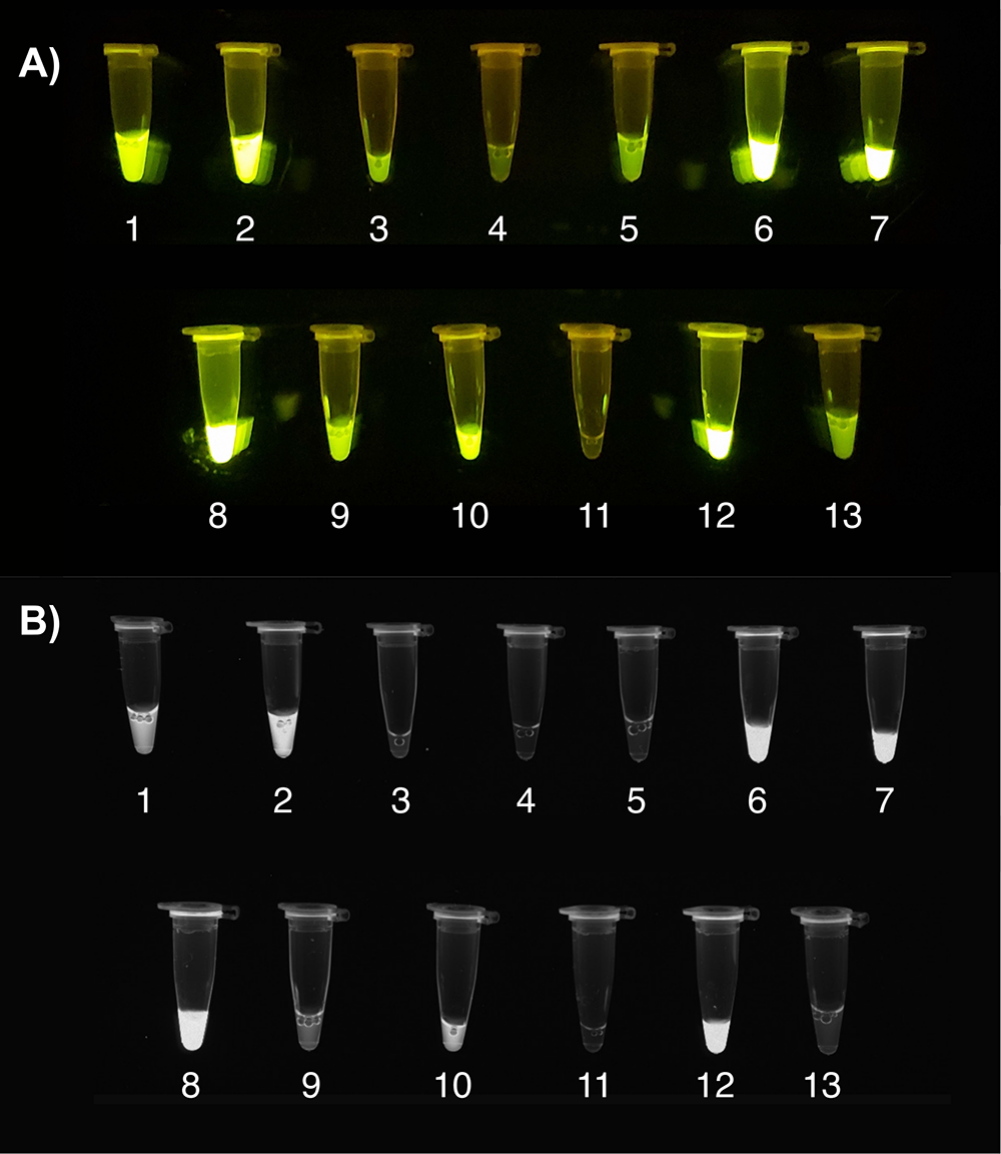
Visual comparison of each condition tested in the first definitive screening design. The numbers represent each condition from Table 3. (A) On a blue-LED transilluminator. (B) Under UV light.

A similar screening strategy was also employed for a one-pot RPA-CRISPR reaction using a DNA template instead of the RNA template. This study aimed to determine differences in the optimal conditions for the one-pot RT-RPA-CRISPR and RPA-CRISPR methods and to expand the optimization of this CRISPR-based one-pot detection method for DNA virus applications. As reverse transcription does not occur in the RPA-CRISPR reaction, factors corresponding to reagents involved in this step (dNTP mix, RNase inhibitor, and RT buffer) were omitted from the DSD. 100 copies/μL 300 bp DNA fragment of N gene (Table S1) was used as template in each condition, and the reactions were incubated for 60 min. Table 3 shows the factors, conditions, and responses (RFU) for the DSD used to optimize the RPA-CRISPR method. The tubes corresponding to each of the different test conditions were also visualized using a blue-LED transilluminator and under UV light as shown in Figure 6.

**Table 3:** Experimental Design and the Fluorescence Responses (RFU) Obtained from the Definitive Screening Design (DSD) Carried out for the RPA-CRISPR Method

condition	reaction volume (μL)	Cas12a/gRNA (nM)	reporter DNA (μM)	temperature (°C)	relative fluorescence unit (RFU)
**1**	50	25	10	42	**133 083**
**2**	50	1000	10	37	**197 276**
**3**	20	1000	0.2	37	**14 243**
**4**	35	25	0.2	37	**3650**
**5**	50	512.5	0.2	42	**11 911**
**6**	35	512.5	5.1	39.5	**345 554**
**7**	20	512.5	10	37	**526 751**
**8**	35	1000	10	42	**1 095 159**
**9**	50	25	5.1	37	**55 320**
**10**	20	25	10	39.5	**114 859**
**11**	20	25	0.2	42	**1199**
**12**	20	1000	5.1	42	**396 128**
**13**	50	1000	0.2	39.5	**19 503**

**Figure 7:**
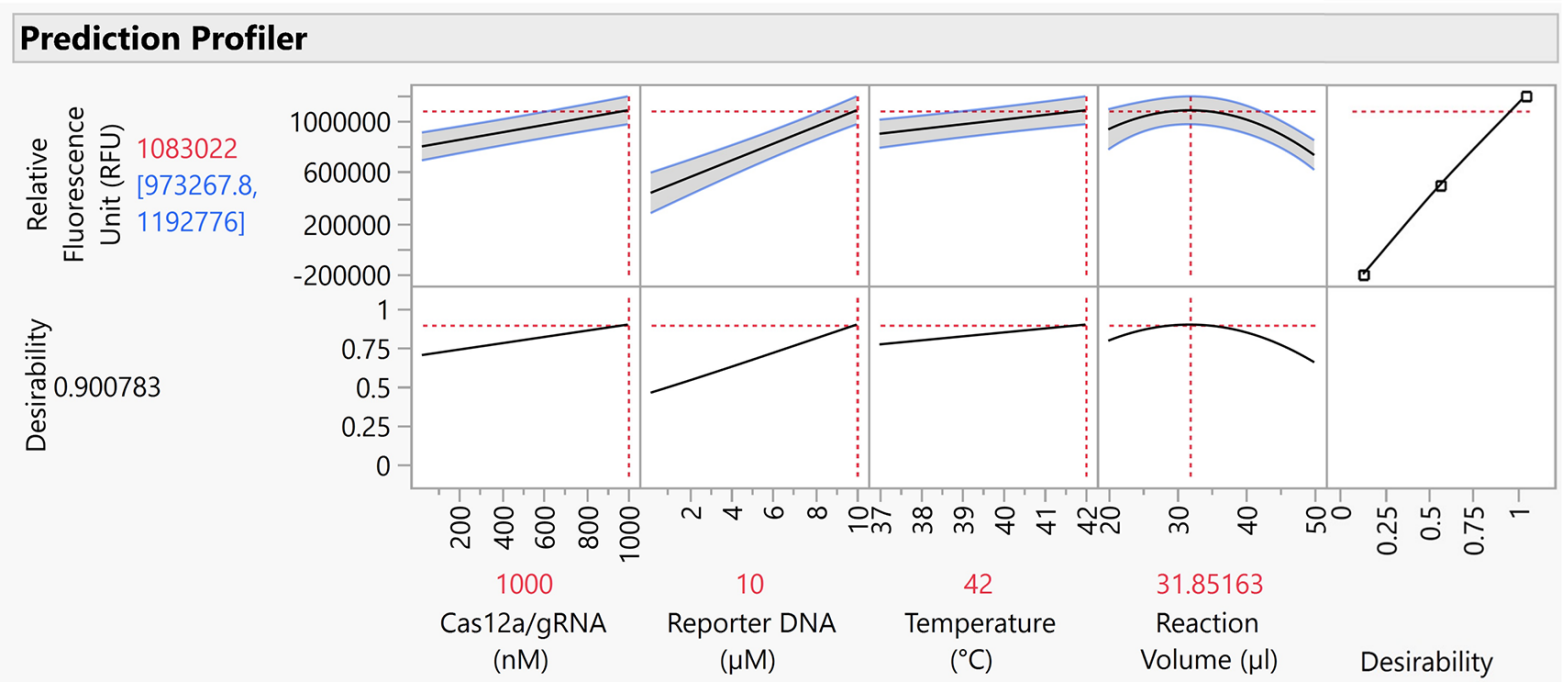
Models shown in the prediction profiler of the software (JMP). The substantial factors and their effects are shown with the maximized desirability score (0.90), reflecting the optimum parameters to obtain the highest fluorescent signal (RFU). The response (or desirability) is shown on the *Y*-axis and the factors are shown on the *X*-axis. The blue numbers shown on the response represent the minimum and maximum responses that can be obtained with the optimum parameter of each factor, while the red number represents the mean of the blue numbers. The gray areas between the blue lines represent the confidence interval for each plot. The plots at the bottom show the maximum desirability when the optimum parameter of each factor is used.

Having a linear relation with the response (Figure 7), Cas12/gRNA complex showed a similar effect on the fluorescence signals as for the RT-RPA-CRISPR method. This is not surprising, as Cas12a activity is the driving force for the creation of fluorescent signals. The fluorescent reporter DNA also showed a linear relationship with the response in RPA-CRISPR (Figure 7), whereas, for the RT-RPA-CRISPR (Figure 4), a plateau was observed at a concentration of around 8 μM. This suggests that the saturation concentration of reporter DNA is higher than the upper limit of 10 μM in the case of the one-pot RPA-CRISPR reaction.

The optimum reaction temperature was observed as 42 °C for RPA-CRISPR (Figure 7), whereas variations in temperature between 37 to 42 °C had no significant effect when it was coupled with reverse transcription. Also, the optimal reaction volume was found to be ∼32 μL, with a nonlinear relationship observed (Figure 7). This also differed from the RT-RPA-CRISPR method, where higher reaction volumes were favorable (Figure 4). DNA templates have been used in some studies in place of the RNA templates^16,22,47^ for optimization of one-pot COVID-19 detection methods. However, one of the most important findings of this study was that the optimal conditions for tests using RNA and DNA templates can differ due to the additional reverse transcription step, especially with the addition of the corresponding buffer, changing the reaction dynamics and conditions. Such differences highlight that the optimum parameters are highly process-dependent and the reactions and conditions of each individual process should be cumulatively assessed. The definitive screening was demonstrated as a valuable tool for the efficient determination of optimal parameter settings allowing rapid development of DNA virus-specific testing methods.

### The Sensitivity of the One-Pot Reaction with Optimized Parameters

The parameters suggested by the models were used to detect the lowest copy numbers of the target nucleic acids for both RT-RPA-CRISPR and RPA-CRISPR methods. However, some factors were kept at the lowest possible value to avoid high detection costs. When preparing the reaction mixes, the Cas12a/gRNA complex (10% of the reaction volume) was mixed with the solution containing all other factors including the templates. For RT-RPA-CRISPR, the following parameters were used:

- 10 U/μL reverse transcriptase, M-MuLV
- 0.5× reverse transcription buffer
- 0.8 U/μL RNase inhibitor
- 1000 nm Cas12a/gRNA complex
- 8 μM reporter DNA (ssDNA F-Q reporter)
- 50 μL reaction volume

**Figure 8:**
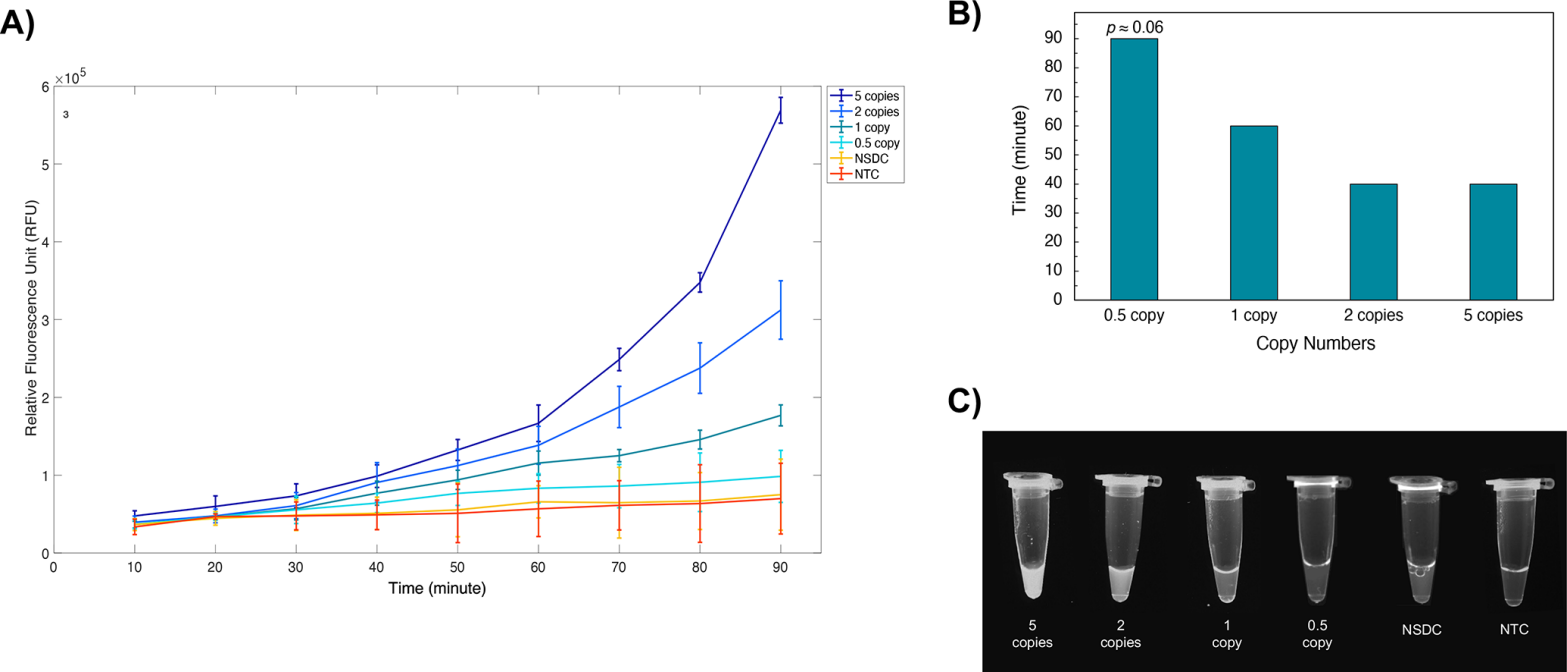
Sensitivity of the optimized RT-RPA-CRISPR. (A) The kinetic measurements of fluorescence intensity (RFU) for different copy numbers and the controls. (B) Minimum reaction times at which a statistically significant difference (*p* < 0.05) from controls (NSDC, NTC) was observed for each copy except for 0.5 copy (*p* ≈ 0.06). The times shown apply to each replicate used for the corresponding copy number. (C) The brightness of the tubes under UV light (only one replicate is shown). NSDC: nonspecific DNA control (contains a yeast plasmid), NTC: nontemplate control.

Although the second DSD revealed that increasing the reverse transcriptase concentration and reaction volume were favorable for high fluorescence yields, the values of these factors were not further increased. This enzyme is one of the most costly reagents in the reaction, and increasing the volume further would increase the amount/cost of all reagents, which is undesirable for low-cost diagnostics. Similarly, the concentration of the Cas12a/gRNA complex was not increased in either of the DSDs or in the sensitivity experiments due to its high cost. The reactions were incubated at 39 °C for 90 min, as the temperature was not a critical factor for the RT-RPA-CRISPR method. The lowest copy numbers of the full-length SARS-CoV-2 genome detected by the optimized method are shown in Figure 8.

The lowest copy number of the full-length SARS-CoV-2 genome which was detected by using the plate reader was just one copy/μL (*p* < 0.05) after 60 min of incubation for all the replicates (Figure 8B). Two copies of the full-length SARS-CoV-2 genome were also detected using a plate reader (*p* < 0.05) and visually using UV light after 40 min (Figure 8B) and 90 min of incubation for all the replicates (Figure 8C), respectively. Statistically, no significant difference was observed between the nonspecific DNA control (NSDC), containing an 11 kb yeast plasmid (p426_Cas9_gRNA-ARS511b) as a nonspecific DNA template, and a nontemplate control (NTC). Although the difference between the 0.5 copy and NSDC was not significantly significant, the *p*-value was only slightly higher than the threshold (*p* ≈ 0.06), which is a promising result for such an extremely low copy number (<1 copy/μL). Compared to similar studies,^15,16^ statistical optimization of the parameters was found to yield a substantial improvement in sensitivity. Further improvements could potentially be achieved if higher Cas12a/gRNA complex and reverse transcriptase concentrations were used along with a higher reaction volume, considering the results of the statistical models (Figure 4, Figure 5). However, as this increases the detection cost, which is a critical parameter for high-throughput testing, these conditions were not implemented in this study.

To determine the lowest detectable copy number of the DNA template, the following parameters suggested by the models were used in the RPA-CRISPR method:

- 1000 nm Cas12a/gRNA complex
- 10 μM reporter DNA (ssDNA F-Q reporter)
- 30 μL reaction volume
- 42 °C reaction temperature

**Figure 9:**
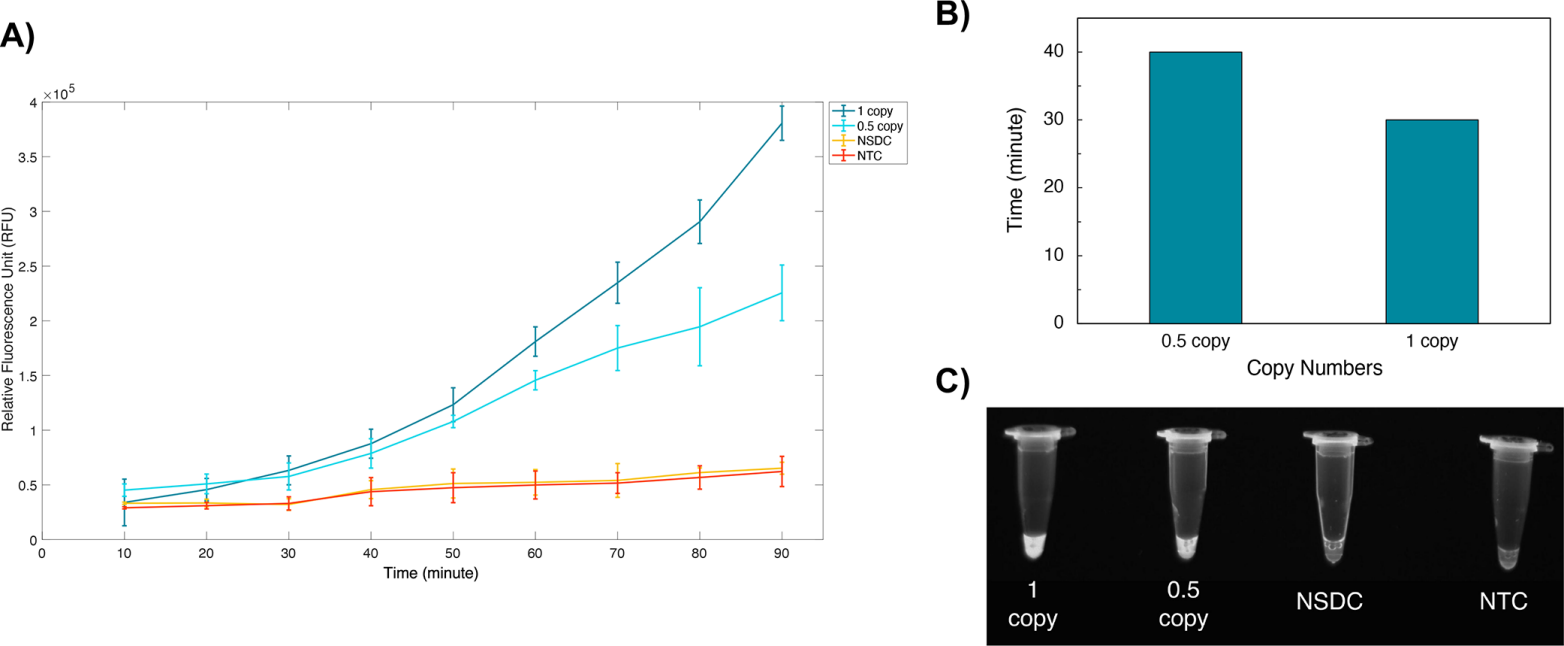
Sensitivity of the optimized RPA-CRISPR. (A) The kinetic measurements of fluorescence intensity (RFU) for different copy numbers and the controls. (B) Minimum reaction times at which a statistically significant difference (*p*-value < 0.05) from controls (NSDC, NTC) was observed for each copy. The times shown apply to each replicate used for the corresponding copy number. (C) The brightness of the tubes under UV light (only one replicate is shown). NSDC: nonspecific DNA control (contains a yeast plasmid), NTC: nontemplate control.

As the RPA-CRISPR method targeting a DNA template has a less crowded environment compared to RT-RPA-CRISPR, higher sensitivity was obtained with 0.5 copy/μL (*p* < 0.05) in 40 min for all replicates using a plate reader (Figure 9B). UV light was also used after 90 min of incubation to visually observe the positive tubes (Figure 9C). This shows that the optimized assay has great potential for one-pot molecular detection of DNA viruses. It also proves that the yield of the reverse transcription is critical for the sensitivity of the RT-RPA-CRISPR method. On the other hand, an increase in RFU over time was observed in NSDC and NTC in both RT-RPA-CRISPR (Figure 8A) and RPA-CRISPR (Figure 9A). This was likely caused by the nature of the ssDNA F-Q reporter. Yet, it might be a problem for the specificity of this method, as Ding et al. (2020) reported a nonspecific increase in fluorescence intensity when a particular Cas12a/gRNA complex was used for the AIOD method.^16^ For this reason, the cause behind this nonspecific increase should be elucidated or further improvements should be made for the specificity to get this method approved for field use.

It should be noted that the ultralow copies were detected under optimum conditions. Even though the nucleic acid samples were only a few microliters, the same volume of clinical samples containing impurities or other chemicals could affect the reaction dynamics. Nevertheless, the optimized condition should be able to detect early stage infections.

While the detection workflow used can be readily automated, commercially available reagents were used for optimization in this study. Among them, the proteins such as Cas12a, reverse transcriptase, and RNase inhibitor were the main reagents increasing the detection cost. On the other hand, high copy bacterial expression vectors with purification tags that are available in Addgene (#102566, #113431, #153314) for these proteins can be used to produce and purify the proteins involved in RT-RPA-CRISPR for high-throughput testing. Currently, there is only one supplier for RPA kits as it is a patented technology.^48^ Although this might be a potential problem for increased demand, the patent will expire in April 2023,^48^ which could allow more affordable alternatives using the same technology to become available in the market in the near future.

## Conclusion

One-pot reactions working under isothermal conditions are promising methods for nucleic acid detection and the molecular diagnosis of infections. Although many efficient one-pot detection methods have been reported, finding the optimal process conditions for these methods can be challenging due to their complex nature. Strategic design of experiments (DoE) approaches facilitate the efficient elucidation of factors with a significant effect on the response and determination of their optimum settings in complex systems in a fraction of the number of test runs, compared to traditional one-factor-at-a-time (OFAT) experimentations. Capitalizing on these DoE benefits in this study allowed the rapid optimization of a one-pot RT-RPA-CRISPR COVID-19 detection method. It was elucidated that reverse transcription buffer and RNase inhibitor, components that are generally neglected in one-pot reactions, increased performance significantly, and optimization of reverse transcription had a critical effect on the sensitivity of the method. Interestingly, the optimum conditions for DNA targeting and RNA targeting methods were found to be distinct, highlighting the importance of testing and optimizing methods targeting different nucleic acid templates separately. By using the optimal factor settings elucidated via statistical modeling, 2 copies/μL of full-length COVID-19 genome and 0.5 copy/μL of DNA fragment of N gene were visually detected. The sensitivity could be further improved by increasing the detection cost as suggested by the models, or additionally, the factors in the RPA reaction could be further optimized since a standard RPA protocol was used in this study. Similarly, further studies might focus on different ranges of the factors such as lower reaction temperatures or volumes to determine the optimum parameters to decrease the detection cost further. In addition, as the one-pot reactions were carried out in an automation-compatible manner, this assay has great potential to abolish manual interventions during the incubations and facilitate high-throughput screening using relatively low-cost automation platforms. Apart from these, the detection capacity of the amplification-free method consisting of two separate reactions, reverse transcription and CRISPR/Cas12a assay, were also sought. However, the specificity of this strategy was not sufficient to be used as a molecular diagnosis technique. Further studies could focus on developing ultrasensitive and amplification-free methods to eliminate the use of extra reagents/reactions and to lower the detection costs. In conclusion, simple and effective detection methods can be optimized by employing DoE so that ultralow copy numbers of target nucleic acids can be detected for early stage diagnostics for high-throughput testing at points of care.

## Supporting Information Available

All sequences and fragments used in this study (Table S1); the comparison of the RPA amplicons produced using different primers (Figure S1); the concentration of the RPA amplicons after purification (Figure S2); the substantial factors and their optimum parameters for the second round of definitive screening design (DSD) for RT-RPA-CRISPR when the AICc stopping rule is used (Figure S3) (PDF)
