# Supplementary material for: Rational Design of CRISPR/Cas12a-RPA Based One-Pot COVID-19 Detection with Design of Experiments
Source: ACS Synth Biol. 2022 Apr 1;11(4):1555–67. doi: 10.1021/acssynbio.1c00617 (PMC9016756; doi:10.1021/acssynbio.1c00617)
Supplement: Supplementary file 1 — sb1c00617_si_001.pdf [file sb1c00617_si_001.pdf]

Supporting Information

Rational Design of CRISPR/Cas12a-RPA Based One-pot Covid-19 Detection with Design of Experiments

Koray Malcı[1,2], Laura E. Walls[1,2], Leonardo Rios-Solis[1,2,3]*

1- Institute for Bioengineering, School of Engineering, University of Edinburgh, Kings Buildings, Edinburgh, EH9 3BF, United Kingdom;
2- Centre for Synthetic and Systems Biology (SynthSys), University of Edinburgh, Kings Buildings, Edinburgh, EH9 3BD, United Kingdom;
3- School of Natural and Environmental Sciences, Newcastle University, Newcastle upon Tyne NE1 7RU, UK

* *Corresponding author:* Dr. Leonardo Rios Solis (Email: leo.rios@newcastle.ac.uk)

Table S1: All sequences and strands used in the study

Name	Sequence (5' – 3')	Reference
Forward RPA Primer (N1)	AGGCAGCAGTAGGGGAACTTCTCCTGCTAGAAT	(1)
Reverse RPA Primer (N1)	TTGGCCTTTACCAGACATTTTGCTCTCAAGCTG	(1)
gRNA1 targeting N gene*	uaauuucuacuaaguguagauCAUCACCGCCAUUGCCAGCC	(1)
gRNA2 targeting N gene*	uaauuucuacuaaguguagauUUGCUGCUGCUUGACAGAUU	(1)
ssDNA-FQ Reporter	56-FAM/TTATT/3IABkFQ	This study
Synthetic 300 bp DNA fragment of N gene	AAGGCTTCTACGCAGAAGGGAGCAGAGGCGGCAGTCAAGCCTCTTC TCGTTCCTCATCACGTAGTCGCAACAGTTCAAGAAATTCAACTCCAG GCAGCAGTAGGGGAACTTCTCCTGCTAGAATGGCTGGCAATGGCGG TGATGCTGCTCTTGCTTTGCTGCTGCTTGACAGATTGAACCAGCTTG AGAGCAAAATGTCTGGTAAAGGCCAACAACAACAAGGCCAAACTGTC ACTAAGAAATCTGCTGCTGAGGCTTCTAAGAAGCCTCGGCAAAAACG TACTGCCACTAAAGCATACA	This study
Forward RPA test primer (N2)	CAAGCCTCTTCTCGTTCCTCATCACGTAGTCGC	This study
Reverse RPA test primer (N2)	CCGAGGCTTCTTAGAAGCCTCAGCAGCAGATTTC	This study

* Lowercase letters represent scaffold sequence

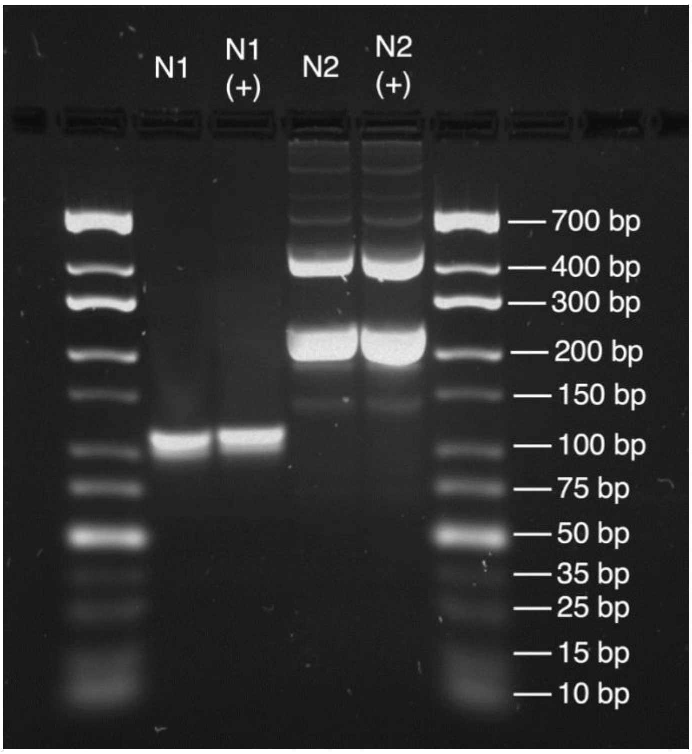

Figure S1: RPA bands on 4% agarose gel before purification. TriDye™ Ultra Low Range DNA Ladder (NEB) was used for the reference bands. N1 fragment was amplified using Forward and Reverse RPA primers targeting 121 bp on the N gene, while the N2 fragment was amplified using Forward and Reverse RPA test primers targeting 237 bp on the N gene. The plus sign (+) represents an additional mixing step during incubation

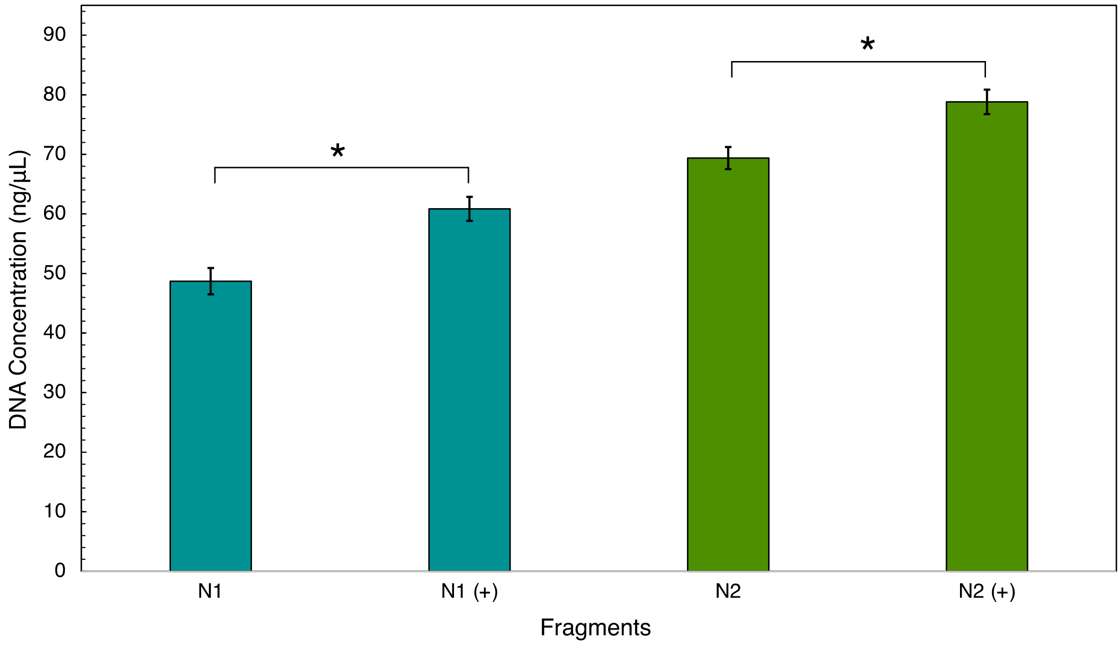

Figure S2: The concentration of the fragments after column purification. N1 fragment was amplified using Forward and Reverse RPA primers targeting 121 bp on the N gene, while the N2 fragment was amplified using Forward and Reverse RPA test primers targeting 237 bp on the N gene. The plus sign (+) represents an additional mixing step during incubation. The asterisk (*) represents a *p*-value < 0.01

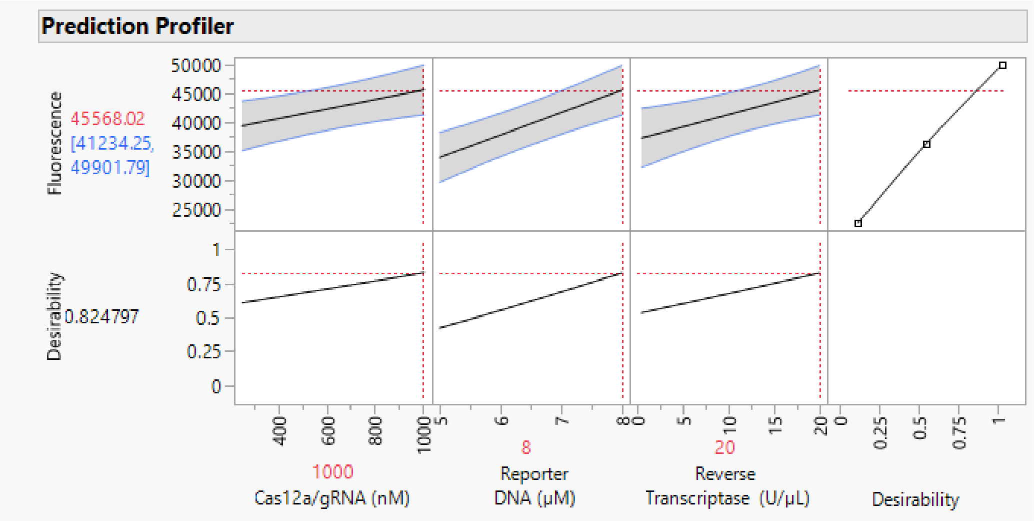

Figure S3: The models shown in the prediction profiler of the software (JMP) with the AICc [2] stopping rule for the second round of definitive screening design (DSD) for RT-RPA-CRISPR. The substantial factors and their effects are shown with the maximized desirability score (0.82), reflecting the optimum parameters to obtain the highest fluorescent signal (RFU). The response (or desirability) is shown on the Y-axis, and the factors are shown on the X-axis. The blue numbers shown on the response represent the minimum and maximum responses that can be obtained with the optimum parameter of each factor, while the red number represents the mean of the blue numbers. The grey areas between the blue lines represent the confidence interval for each plot. The plots at the bottom show the maximum desirability when the optimum parameter of each factor is used.

References

(1) Ding, X.; Yin, K.; Li, Z.; Lalla, R. V.; Ballesteros, E.; Sfeir, M. M.; Liu, C. Ultrasensitive and Visual Detection of SARS-CoV-2 Using All-in-One Dual CRISPR-Cas12a Assay. *Nat. Commun.* **2020**, *11* (1), 1–10. https://doi.org/10.1038/s41467-020-18575-6.

(2) Posada, D.; Buckley, T. R. Model Selection and Model Averaging in Phylogenetics: Advantages of Akaike Information Criterion and Bayesian Approaches Over Likelihood Ratio Tests. *Syst. Biol.* **2004**, *53* (5), 793–808. https://doi.org/10.1080/10635150490522304.
